# Under the banyan tree - exclusion and inclusion of people with mental disorders in rural North India

**DOI:** 10.1186/s12889-015-1778-2

**Published:** 2015-05-01

**Authors:** Kaaren Mathias, Michelle Kermode, Miguel San Sebastian, Mirja Koschorke, Isabel Goicolea

**Affiliations:** Landour Community Hospital, Landour, Uttarakhand 248179 India

**Keywords:** India, Exclusion, Stigma, Inclusion, Mental illness, Qualitative

## Abstract

**Background:**

Social exclusion is both cause and consequence of mental disorders. People with mental disorders (PWMD) are among the most socially excluded in all societies yet little is known about their experiences in North India. This qualitative study aims to describe experiences of exclusion and inclusion of PWMD in two rural communities in Uttar Pradesh, India.

**Methods:**

In-depth interviews with 20 PWMD and eight caregivers were carried out in May 2013. Interviews probed experiences of help-seeking, stigma, discrimination, exclusion, participation, agency and inclusion in their households and communities. Qualitative content analysis was used to generate codes, categories and finally 12 key themes.

**Results:**

A continuum of exclusion was the dominant experience for participants, ranging from nuanced distancing, negative judgements and social isolation, and self-stigma to overt acts of exclusion such as ridicule, disinheritance and physical violence. Mixed in with this however, some participants described a sense of belonging, opportunity for participation and support from both family and community members.

**Conclusions:**

These findings underline the urgent need for initiatives that increase mental health literacy, access to services and social inclusion of PWMD in North India, and highlight the possibilities of using human rights frameworks in situations of physical and economic violence. The findings also highlight the urgent need to reduce stigma and take actions in policy and at all levels in society to increase inclusion of people with mental distress and disorders.

## Background

People with mental disorders (PWMD) are among the most marginalised in all societies with narratives of social exclusion across nearly all cultures. The World Health Organisation (WHO) defines social exclusion as “the dynamic, multi-dimensional processes driven by unequal power relationships interacting across four main dimensions - economic, political, social and cultural - and at different levels including individual, household, group, community, country and global levels”. Social exclusion therefore creates a sense of not belonging, non-participation in community life and diminished opportunity and capacity to participate. It results in unequal access to resources, capabilities and rights which leads to health inequalities [1].

Stigma is a key driver of social exclusion. Stigma occurs when labelling, status loss, stereotyping, separation and discrimination occur together in situations that allow them [2] clarifying that social exclusion entails both prejudice (attitudes) and discrimination (behaviour). It leads to individuals being perceived as ‘tainted and discounted’ [3]. Social exclusion can be conceptualised as interlocking and mutually compounding problems of impairment, discrimination, diminished social role, lack of economic and social participation and disability [4].

Mental illness is complexly related with many components of social exclusion such as lack of social networks, underemployment [4]. Exclusion of PWMD contributes to under-treatment, social isolation, low help-seeking and limited access to care [5] leading to a vicious circle of further exclusion [6]. When working with communities to support and build mental health, understanding the mechanisms of exclusion and inclusion and the experiences of PMWD informs the development of effective responses to reduce stigma, improves access to care, and amplifies ‘safe social spaces’ [7].

In this paper we define social inclusion in relation to mental health as “a virtuous circle of improved rights of access to the social and economic world, new opportunities, recovery of status and meaning, and reduced impact of disability” [4]. Social inclusion includes the opportunity and ability to participate as one wishes, to exercise the rights and responsibilities of full citizenship [8]. In seeking social inclusion, PWMD are better conceived as agents than ‘consumers’, [9] for whom recovery includes recovery from experiences of social exclusion.

WHO and mental health programmes in high income countries (HICs) increasingly recognise the importance of social inclusion as a key outcome for evaluating the effectiveness of mental health programmes. Inclusion and exclusion can be conceptualised as an interwoven continuum. Though critically important there are sparse definitions and surprisingly little understanding of how to integrate inclusion into practice particularly in low and middle income countries (LMICs) [10,11].

### Social exclusion of PWMD in North India

India, a vast country, with one seventh of the world’s population is home for millions of PWMD. The urgent need for greater understanding of exclusion and how it impacts access to care has been highlighted [12,13]. Up to 90% of people in India with mental disorders do not have access to bio-medical care [14]. The majority of studies which describe experiences of exclusion for PWMD, have been conducted in South India [13,15-19], and East India [20,21]. Almost no research has described experiences of exclusion of PWMD in the Hindi speaking belt representing perhaps 600 million people.

Important features in the landscape of PMWDs stigma experiences in India show exclusion as widely experienced and also moderated by gender and age [15-17,21,22]. South Indian studies showed that not only were women with mental illness stigmatised in relation to marriage, but a woman’s illness also prejudiced marriage opportunities for relatives [13,23]. These results underline the gendered role definitions in Indian society as well as the halo effect of stigma, i.e. the excluding social processes such as reduced marriage opportunities for female relatives, that impact whole families.

A recent literature review focussing on social inclusion/exclusion of PWMD found all 36 studies came from High Income countries (HICs) [10]. The urgent need for qualitative and contextually located accounts of stigma, inclusion and exclusion in global mental health research has been acknowledged [24,25]. Quantitative methods can miss textured nuances of experience and narratives of inclusion that are concomitantly present [26,27].

This research formed part of the baseline data collection for two community mental health programmes, and the findings are being used to inform initiatives that will promote social inclusion of PWMD.

## Methods

### Setting

We conducted this research to understand exclusion and inclusion of PWMD in two rural communities set in Seohara and Sadoli-Kadim, two administrative blocks of western Uttar Pradesh (UP), India. We worked with two project teams of Emmanuel Hospital Association (EHA) (www.eha-health.org), an organisation that works across North India in community health and development. These teams had been focussing on community mental health promotion since April 2012 (Saharanpur district) and April 2013 (Bijnor district). Western UP has been a recent flashpoint for communal violence with riots and protests in both 2013 and 2014. Saharanpur is a relatively poor district with 3.5 million people (40% Muslim, 60% Hindu) and a literacy rate of 70%. Health indicators for the district are lower than national and UP state averages. Bijnor district has 3.6 million people (66% Muslim and 32% Hindu) and a literacy rate of 78% [28].

The national District Mental Health Plan was re-launched in India in 1996, seeking to ensure that there is a psychiatrist and psychologist as part of a mental health team for each district. It has been imperfectly and incompletely implemented across the country [29,30]. In both study districts, there were no psychologists, no government mental health services and only one private psychiatrist. PWMD typically visit local ‘doctors’ (no formal training) and traditional religious healers.

Both study areas are dominated by agricultural land dotted with large villages of 1000 – 8000 people, with densely co-located houses. The majority of families own their house but work as labourers for large land owners. In a typical rural UP household, the eldest son and his wife and family, and other sons, live in a joint family with the mother and father. Daughters move into the family home of their husbands after marriage. Family resources for inheritance are divided among the sons in both Muslim and Hindu communities.

### Participants

EHA community workers identified known community members as potential participants fulfilling the following inclusion criteria: A with a likely mental disorder; B greater than 17 years of age; C willing to participate in this study and D living with family members/ a caregiver. The first author (a female medical doctor) screened these potential participants for a mental disorder using the Global Mental Health Assessment Tool [31]. 20 PWMD/primary caregiver dyads (pairs) were identified who met criteria for a mental disorder and who consented to be interviewed for this study. 19 of 20 PWMD experienced severe mental disorders (E.g. schizophrenia or post-partum psychosis).

The majority of PWMD had never visited a mental health service provider and had no psychiatric diagnosis. Only two were taking psychotropic medication at the time of interview. Seven (of 20) PWMD were too unwell to contribute to interviews (i.e. they were psychotic, non-responsive and unable to concentrate) so interview data from their 7 caregivers was included in analysis. Of the 13 PWMD well enough for interview, eight were interviewed separately from their caregivers. These interviews did not reveal data importantly different to those conducted with both caregiver and PWMD present. At five interviews there were additional observers of the interview, each was a direct relative and there by request of the participants. Names used in this manuscript are fictitious.

### Data collection

A female social worker research assistant conducted the interviews in June 2013 in the homes of participants. She had worked several years in the study area, and had been trained to conduct in-depth qualitative interviews. The first author who is trained and experienced in qualitative research methods, supervised the first four interviews and performed follow up interviews with three participants at a later date. The interviewer used a semi-structured in-depth interview guide used elsewhere to investigate stigma of people with schizophrenia in India [13]. The interview guide included open-ended questions about experiences of mental disorders, including help seeking behaviour /participation, and experiences of inclusion and exclusion. Interviews were recorded verbatim with a digital voice recorder, then listened to repeatedly, translated and transcribed from Hindi to English using word processing software by the research assistant. Interviews typically lasted from 45 to 90 minutes.

### Analysis

The interviews were analysed using primarily an inductive approach while acknowledging the theoretical assumptions within in the study design, the initial research questions as well as existing knowledge which influenced the topics covered by the interview guide. Qualitative content analysis was chosen as an approach that is attentive to both manifest and latent content of data [32]. KM read and re-read the transcripts of the interviews. Meaning units describing exclusion and inclusion of PWMD, driven by the data, were identified and coded. Emerging codes were discussed by authors (KM, MKe and IG) and grouped to form categories. Finally, cross-cutting themes were sought, to make explicit latent content in the text. Open Code software was used to help in the coding process [33]. Table 1 provides an example of the coding process.

**Table 1:** **Example of coding process**

**Selected codes**	**Categories**	**Theme**
Choosing not to visit household since X became sick	Loss of relationship due to illness	Distancing
Neighbours no longer visiting this house		

### Ethics

Informed consent, compliant with ethical guidelines on informed consent for PWMD [34,35], was obtained. All potential participants who were mentally unwell at the time of screening were given information on how to access psychiatric care. The study was approved by the Emmanuel Hospital Association Institutional Review Board of Ethics in April 2013.

Table 2 summarises socio-demographic characteristics of participants.

**Table 2:** **Profile of 20 PWMD and 8 caregiver participants**

**Variable**	**Detail**	**PWMD**	**Caregiver**
**Sex**	Women	9	5
	Men	11	3
**Age**	Range	22 to 50 years	25 to 50 years
	Mean age	38 years	39.5 years
**Religion**	Muslim	9	3
	Hindu	11	5
**Employment**	Government health worker	1	0
	Responsible for house	7 of 9 women	5 of 5 women
	Daily wage work	4 of 11 men	3 of 3 men

Table 3 in the presents participants directly quoted in this paper.

**Table 3:** **Participants cited in this paper**

**Pseud-onym**	**Age**	**Sex**	**Possible mental illness**	**Religion**	**Household situation**
Raju	45	M	Bipolar disorder	Hindu	Lives with wife and 2 adult children
Firoz	42	M	Schizophrenia	Muslim	Lives with brother Faiz and his family
Faiz	48	M	-	Muslim	Brother of Firoz, lives with wife and children
Nilofer	35	F	Post-partum psychosis	Muslim	Lives with husband and 5 children under 10 years
Perez*	28	M	Bipolar disorder	Muslim	Lives with wife and their children in his parent’s home, and sister Pakeeza
Pakeeza	25	F	-	Muslim	PCG, lives with parents and 11 siblings and their families, including Perez
Anju	28	F	Post-partum psychosis	Hindu	Lives with husband and 2 children
Golu	35	F	Schizophrenia	Muslim	Single woman head of household, 4 children, husband working in Delhi
Kundan*	24	M	Schizophrenia	Hindu	Lives with parents Kartik and Kavita, and brother
Kartik	50	M	-	Hindu	PCGs, live with Kundan and one other son
Kavita	49	F	-	Hindu	
Pravin*	24	M	Schizophrenia	Hindu	Lives with mother Preeti and 2 siblings
Preeti	50	F	-	Hindu	PCG, widowed, lives with Pravin and 2 other children
Jitin*	45	M	Schizophrenia	Hindu	Lives with wife, Jyoti and 4 children
Jyoti	45	F	-	Hindu	PCG, lives with husband Jitin, and 4 children
Lata	45	F	Post-partum psychosis / depression	Hindu	Lives with husband and children, including 2 married sons and their wives
Shmayla	32	F	Post-partum psychosis	Muslim	Lives with husband and 3 children with her sisters also living nearby

“*” indicates person with mental disorder who was too unwell to contribute to interview and therefore his/her primary caregiver (PCG) data was also used in analysis.

## Results and discussion

12 key themes emerged from the data. Under the sub-heading of exclusion fall the themes of Distancing, Disregarding, Negative judgement, Social isolation, Minimising discrimination, Unworthiness, Verbal violence, Economic violence and Physical violence. While experiences of exclusion were dominant for nearly all participants, the sub-heading of inclusion includes three themes, Participation, Belonging and Support related to experiences of inclusion showing experiences to have a dappled light mixed through the darkness of exclusion. Finally experiences of exclusion and inclusion can occur together, as described under the last sub-heading.

### Exclusion

#### Distancing

Since they had become mentally unwell, PWMD felt people were less willing to interact with them. In their communities, friends and neighbours would sometimes not acknowledge them and rarely sat to talk as they had done earlier. Raju (45 years, man) described this sense of distancing also occurring in his relationships at home:

*Not just outsiders but my family also maintained distance…..even my wife and children. I feel bad still as my children do not talk to me. I feel hurt when my children do not come to me often.*

Participants described direct interactions with neighbours diminishing after onset of the illness and neighbours talking *about* the person affected, but no longer interacting *with* them. Faiz (45 years, man), describes the loss of social interactions for his brother Firoz:

*No friend of his has ever come to see him. When he goes out he recognizes people, but people do not talk to him. People say he is mad and there is no use talking to him… No one actually hates my brother, but I wish that people would stop pitying him. People keep saying, ‘Poor fellow what has become of him?’ But no one says anything to him at all, and instead they talk about him amongst themselves.*

We often observed family members talking on behalf of the affected person in their presence, without giving them any opportunity to speak. However, it is important to note that unwell PWMD, often with untreated chronic illnesses, were almost mute, and several household members said that the affected person had only spoken a few words in many months.

### Disregarding

Exclusion from social interactions extended to disregarding PMWD’s voices -e.g. discouraging PWMD participation in family discussions. Even when the PWMD could be authoritative, such as describing their symptoms, some family members disregarded their narratives. Nilofer (35 years, woman), who had had experiences of voice-hearing after her previous delivery, described how she related the recurrence of her voice-hearing symptoms to her husband who disbelieved her:

*Now after my baby boy was born some voices started in my head again. If anyone spoke something I felt that my mind had swollen. I was unable to hear anything and was so angry that I could have either killed myself or someone else. When I told my husband about this, he said that cold and flu has affected my mind. I told him that it is not flu, it’s the same illness that I had long ago. ….*

Very few PWMD were involved in household decision making, often reporting that when trying to contribute, they were told they were incapable. This suggests some PWMD were stripped of their agency, even in their own homes, as Nilofer describes:

*My husband does not tell me when he takes some decision. When I ask him something, he says ‘Why do you need to know? You are mad.’ He says that I try to be smart. I feel very bad when he says so.*

### Negative judgement

Participants described how neighbours negatively judged the behaviour of PWMD, E.g. Pakeeza, (25 years, female) describes neighbours’ judgements of her brother Perez’ behaviour as an elaborate ‘drama’ to evade work:

*Our neighbours say that Perez doesn’t want to work so he is faking all this drama. But actually my brother wasn’t like this at all earlier. Before he became unwell he was a very hardworking person… like I remember when we was working as a truck driver he would drive from Moradabad to Delhi and back to Moradabad again* (8 hours driving) *without stopping for a meal break.*

Significantly caregivers said that negative community responses to PWMD seemed to be greater when the PWMD displayed socially unacceptable behaviours. Caregivers described their family member facing greater ridicule when they disclosed symptoms such as commanding auditory hallucinations, or engaged in unusual behaviour such as collecting rubbish in pockets to take home.

### Social isolation

Participants described erosion of relationships and friendships leading to social isolation. A rather abrupt loss of social connection related to her brother’s mental disorder is narrated by Pakeeza:

*When my brother was fine and worked as a driver, half of the village was jealous. When he used to come home from his job, the environment of the village would become like it was Eid* [Muslim festival celebrated after a month of fasting]*. People appreciated him a lot and were always standing around him in a crowd. My brother was tall and handsome and he had many friends at that time. But now when my brother isn’t well none of his friends come to see him and now they make stories up about him and laugh at him.*

For others, interactions in the community became so painful, that they stayed at home except for required transactions. Family members also exhibited overtly excluding behaviours, as in Nilofer’s account of exclusion from the common family bathroom:

*And my sisters in law didn’t even allow me to bathe in their bathrooms. After my child was born, I had to raise the cot on all sides and take my bath there. (…) And then, when they let me use their bathroom just occasionally, they would wash their bathroom afterwards, as if I had some bad disease. I felt really bad at that time.*

### Minimising negative judgements

Participants often would deny experiences of discrimination while almost simultaneously describing their occurrence. There is a sense of participants wanting to believe they are part of the community despite the facts. Anju (28 years, female) exemplifies this, after the interviewer asked whether people ever made fun of her:

*Nobody said such things and everyone was worried about my health*.[In agitated and louder tone] *Even if 2–3 people said something, we can’t do anything. You cannot seal people’s mouth as they will speak nonsense if they want.*

The following quote from Faiz initially insists that there was no discrimination against his brother in the community, but then immediately describes the loneliness of being a caregiver suggesting that community members’ attitudes would have been more supportive if his brother had a physical illness:

*No the villagers do not discriminate against him or us – my brother Firoz was very good earlier and people know that so no one treats us badly like that. Still I feel alone when no one helps me.*

[Interviewer: Do you think the villagers’ attitudes would have been different if Firoz had an illness like diabetes?]

*Of course, then people’s attitude would have been good.*

### Unworthiness

Several female PWMD believed that family members no longer valued or cared about them. Underneath this was a sense of abandonment and being unvalued as described by 35 year old Golu:

*My husband is not even slightly worried about me. Even if I die he will not come to see me. When I fall sick, he tells my son by phone to get medicines for me rather than going himself. He never cares.* [Long pause] *The thing is, all his brothers listen to their wives, but he doesn’t listen to me. He says that I am mad and after he has had his way with me* [speaking of sexual relations] *he goes away.*

PWMD also described a sense that their value within a household was contingent on remaining mentally healthy. Nilofer said that her sisters-in-law would have thrown her out of the house if her episode of mental illness had persisted:

*Once they said to me ‘If you hadn’t recovered and had remained mad, we wouldn’t have kept you in the house for a single minute’. I feel so sad that when I am fine, I am allowed to remain here but they would have thrown me out when I was not in my senses. Where could I have gone?*

### Verbal, economic and physical violence

Ridicule of PWMD in public settings was a frequent and salient experience described by most participants. Kavita (50 years female), described the public derision experienced by her son.

*Yes, people laugh at my son. Yesterday also someone was mocking him and* [because he believes he has high connections] *saying “Sir, please take whatever you want and rest somewhere. We will get you water.” Villagers very often make fun of my son.*

### Economic violence against PWMD

Jyoti, Preeti and one other female caregiver, each described how, due to their son’s/husband’s mental disorder their nuclear family had been disinherited (i.e. they did not receive their share of the family inheritance). Jyoti, describes her husband Jitin’s disinheritance and virtual excommunication from the family below:

*Since his illness all the times are difficult with us.* [Before] *we lived on the other side of the village with our in-laws and relatives then since his illness we were sent to this place and given this two room house…. All our relatives of my in-laws side have broken all contact with us. They haven’t given him any money or share in the property….*

### Physical violence against PWMD

Overt physical violence against PWMD, carried out by both family members and neighbours, was a frequent experience. Perpetrators recounted their violent acts without prompting, or sense of shame. Family members described ‘beating’ to encourage the affected person to ‘get better’. In some households, family members indicated discomfort with interpersonal violence and tried to intervene in violent family interactions. Pakeeza described her father’s actions towards her brother Perez:

*“How much can he beat my brother when he isn’t improving? My dad tells him to quit bhang* [cannabis] *and my brother refuses to do so. Now we just remain silent but my father beats my brother when he gets angry…. My father also tries his best not to beat him.”*

### Inclusion

#### Opportunity for participation

The majority of participants had no expectation that their mental health status could be concealed yet felt free to at least passively participate in community activities. Most PMWD described freedom to visit neighbours’ houses as they had before their illness, and to attend weddings, social functions and places of worship. As 50 year old Kartik described:

We never felt that we were treated differently. Even Brahmins visit my family and we are involved in every celebration and decisions in our village.

Although the majority of PWMD described exclusion from decision-making, some had opportunities to participate. Lata (45 years, woman) describes her responsibility in relation to household finances:

*My husband took my advice in every matter and most of the decisions were mine. Children’s education, clothes and other expenses of the house were totally my responsibility. ( ) The responsibility for managing the money and running the family was mine and right through my sickness my husband gave me the money to handle.*

### Support

Some PWMD and caregivers described substantial support from neighbours during periods of mental distress, which was interpreted as a marker of acceptance and belonging. Support was described in two main forms: firstly, others taking responsibility for the PWMD and caregivers’ normal responsibilities, and secondly, accessing resources to take the PWMD to health and healing services.

Practical support took many shapes and included feeding and caring for the PWMD and /or caregivers’ children, helping with housework and harvesting, lending money for health costs, and providing support to caregivers. There were many examples of caregivers performing tasks outside those typically assigned by gender roles. Here, Anju’s husband describes doing tasks usually assigned to women of the household as well as the community’s support:

*At times I did all the house work or some relatives of ours helped in the household work….. In villages the good thing is that if there is trouble at one person’s home, the neighbours will collectively complete their work. People from our village also helped us a lot when she was ill.*

PWMD described the significant expenditure and effort that caregivers had made to seek care for them as evidence of being valued. Several households had to sell land or livestock to pay for care of the PWMD. Anju (28 years, woman) described:

*For poor people harvest is an important thing. But due to my illness fields were left like that and even my cattle also…..A lot of money was spent. A human is more loveable than money. You can raise money, once the person becomes ok. See now when I am ok, that loss of money doesn’t matter to us.*

PWMD described reciprocity as a key driver for neighbourly support. Lata described how during her hospital admissions, when she and her husband were both away, neighbours took her children to school, cared for the family cow and watered their crops, which was related to reciprocity norms within her caste:

*We are Harijan and always help someone who is of our caste. If someone in a family has a seizure and they need help to go to hospital then we will help that person. But it is the kind of helping where they know you also have to help them in their time of need. ( ) even if I take 1 kg of rice then a few days later I must give it back in full to that neighbour.*

### Sense of belonging

Many PWMD experienced ongoing positive relationships with relatives and family members as important for their own wellbeing and sense of belonging. Female PWMD in particular described the importance of their maternal families who in several instances were the primary source of care. Several dyads said their relationships had become stronger during and after the difficult period of mental illness as Nilofer describes:

*Our relationship is better after I fell ill. Now my husband treats me like I have a new life. Earlier my husband never loved me and wasn’t interested in me…. But when I fell ill and he looked after me, he started valuing me and now I am very happy. He says that I have undergone too many difficulties…*

Relationships that were challenging and later became positive were particularly mentioned as meaningful and restorative. Nilofer also acknowledged humour and restoration in her relationship with her aunt:

*My aunt once came to visit me … and I spoke bad words to her. She felt bad and left. …. Now she is ok as she understood that I was not in my senses at that time. Now she rings up and she laughs and asks whether I will behave properly or not when she visits me next time.*

### Exclusion and inclusion

Sometimes experiences of exclusion and inclusion occurred simultaneously for one individual. During her post-partum disorder Nilofer was physically excluded from the common family bathroom, while at the same time increasingly nurtured and cared for by her husband, which she directly attributed to the presence of her mental illness. Preeti described her son Pravin being able to freely worship at the village Hindu temple, yet being called names as he walked there and back. Experiences of exclusion and inclusion for PWMD in these rural Indian communities are complex and entangled.

## Discussion

These narratives present PMWD experiences as predominantly of exclusion yet with strands of inclusion woven through. They exemplify understandings of exclusion as inability to participate in economic, social, cultural and political spheres of life [8].

These accounts of exclusion suggest there is often a limited sense of belonging and perhaps even compromised safety for PWMD in public spaces. While most of the people interviewed were too unwell for regular paid employment, there was little opportunity for any participation in economic and political life. However, there was participation in facets of social and cultural life for PWMD such as attending community functions and places of worship.

Three dimensions of exclusion for PWMD were apparent. Firstly, community members’ negative judgements were in some part fuelled by poor knowledge about mental illness. Narratives of neighbours accusing PWMD of ‘faking’ their illness suggests insufficient knowledge about the nature and presentation of mental illness, and perhaps an unwillingness to try to understand their illness experience. Poor knowledge (also described as low mental health literacy [36]) contributing to stigma has been identified by others in India [16,37,38]. However, it is simplistic to believe that increasing knowledge as a single intervention is enough to reduce the stigma associated with mental illness and the associated exclusion. Negative consequences of attributing behaviours to an ‘illness’ include increased stigma, i.e., even when illness behaviours have abated, the person might still suffer from the label of having ‘an illness’. In fact emerging evidence suggests that stigma reduction interventions promoting a bio-medical model of mental illness may increase rather than decrease stigma [39,40]. More effective stigma reduction interventions include direct contact between stigmatised and stigmatising people, role modelling of acceptance, and involvement of mass media [41,42].

Secondly, distancing behaviours by both community and household members were attributed by PWMD to the stigma associated with their mental illness. However some of this distancing may have been related to PMWD’s own illness behaviours (E.g. non-response to repeated efforts at communication) leading to people feeling afraid or unsure of how to communicate. Other reasons for distancing may have been due to a belief that PWMD were incapable of meaningful social interactions, suggesting again the need to increase mental health knowledge and understanding in communities as well as the importance of access to care, to increase social function of PWMD.

Thirdly, excluding words and behaviours by community members seemed to be greater for people with poor self-care. The majority of PWMD in this study had highly disabling, untreated chronic severe mental disorders. Several of those interviewed engaged in socially unacceptable behaviours such as collecting rubbish in the streets and poor personal hygiene. Others have described higher levels of exclusion related to socially unacceptable behaviours [13,22]. This is a vicious circle: loneliness and social exclusion increase the risk of poor mental health and concomitant behavioural manifestations, which in turn increase social exclusion [6]. In the context of Uttar Pradesh, it is possible that PMWDs experience greater social exclusion because they can’t access treatment for socially stigmatising behaviours. There is an urgent need to increase both awareness and access to care.

It is noteworthy that unworthiness was primarily an experience of female PWMD in this study. Self-stigma is the prejudice that people with mental illness turn against themselves [43] and negative self-regard or unworthiness are augmented by discrimination [44]. Several Indian-based studies have described an association of higher stigma with the female gender [15,16,22] even though women with severe mental disorders typically have better outcomes and prognosis with fewer negative symptoms [45]. A recent large study of exclusion and mental illness in three sites in South India found no quantitative gender differences in the frequency of discrimination, but qualitative differences in the way stigma manifested in men’s and women’s lives [13]. Women’s greater vulnerability to physical and mental illness is apparent in a country where gender-based discrimination, lower income, lower social status and associated factors of poverty, overwork, under-nutrition for women are highly prevalent [46,47]. They sum to high levels of undervaluing and disadvantage for women in India, one of the world’s most gender unequal countries [48].

Minimising experiences of exclusion may be a mechanism to help participants reduce the pain associated with ostracism, and to defend neighbours to an external observer such as the interviewer. It may indicate levels of self-stigma, where a PWMD is more likely to minimise or endorse discrimination by others. Acknowledging the prejudice and negative judgements of neighbours perhaps risks admitting the painful reality of ones’ exclusion.

Verbal violence/ ridicule and an ensuing sense of social ostracism and public judgement were also salient experiences. The overt taunting described in this research is a more intense form of verbal violence than simple name calling. The impact of verbal violence leading to self-imposed social isolation of several PWMD and related household members in this study is an important finding reiterating that exclusion impacts significantly on the whole household.

We have not found other reports of male offspring with mental disorders being ‘disinherited’ in research on exclusion and stigma of PWMD in India. Disinheritance probably reflects the inability of the male family member to claim his share for himself. As financial matters are often discussed with male family members, others take advantage of his psycho—social disability to encroach on his rights. Disinheritance is overt financial exclusion by a family of origin and further setback for families already impoverished by loss of earnings if the affected adult cannot work, as well as costs related to care seeking, and the opportunity costs of care giving.

Accounts of physical abuse and intimate partner violence for PWMD have been widely described in HICs [49-51] and in some LMICs [52]. It was an unexpected theme identified in a qualitative study performed among people with schizophrenia in South India [23] and in a mixed-methods study in South India [13]. It is likely that violence against people with mental illness by family members is highly prevalent across India, yet it has received insufficient mention as a human rights issue. This violent, physical exclusion affects PMWDs health and well-being, likely exacerbating their mental illness, and demands urgent attention and advocacy [53].

Research on social inclusion for PWMD is almost exclusively located in HICs where there is reasonable access to care. This research underlines how critical access to care is if a socially inclusive environment for PWMDs is to be achieved [4]. However, even in this rural North Indian setting, social exclusion is not absolute and this study reports several rich accounts of social inclusion. Baumgartner’s four key features of social inclusion are: a sense of belonging, active participation in community and civic life, a sense of agency and capacity to choose to participate, and opportunity for participation [54]. These features map well onto the themes identified in this study of opportunity for participation, a sense of belonging and support.

Building on Amartya Sen’s “Capabilities” approach to development [55], Ware understands social inclusion as “a process, unfolding over time, through which individuals who have been psychiatrically disabled, increasingly develop and exercise their capacities for connectedness and citizenship.” [9] This definition recognises that social environments must be supportive to PWMD, and builds on a social theory of disability which locates disability in society and identifies disabling structures and policies that exclude affected individuals.

Characteristics of receptive social environments were evident in these rural communities in Western Uttar Pradesh such as opportunities to visit neighbours as before the illness, visits by Brahmin priests, and practical neighbourly support during times of illness. There was also a clear sense that most PWMD had the opportunity to participate in community functions.

Reduced capacity to participate is a key limitations for people with mental illness. All eleven male PWMD in this study were too unwell to sustain regular paid employment indicating the low functional level they were living with. Access to care brings the possibility of relief from stigmatising symptoms of mental illness and can increase social and economic participation [4]. There is an urgent need for better access to care for millions of PWMD across India, and is important if we are to increase social participation and inclusion for PWMD.

### Methodological considerations

To address methodological rigour in this study, we incorporated four strategies to promote the trustworthiness of the findings [56]: credibility, transferability, dependability and confirmability.

Triangulation using different methods, different sites and analysis by different authors with different educational and cultural backgrounds increased the study’s credibility. Two methodological aspects may reduce credibility: Firstly, the involvement of researchers (KM and research assistant) with their recognised links to an established community health programme, may have led to respondents give socially desirable responses. Secondly, by using data from caregivers, we risk portraying caregivers’ rather than PMWD perceptions. Interviewing some PWMD in the presence of caregivers also raises the possibility of social desirability bias. We minimised these concerns by interviewing a relatively large number of people, by privileging PWMD narratives over caregiver narratives, focussing on data that triangulated with PWMD narratives, and looking for discrepancies between PWMD and caregiver accounts. In many aspects data saturation was reached after reviewing the first ten interviews making it less likely that major new themes were omitted through the minimal participation of seven PWMD.

Transferability: We tried to maximise this by providing detailed information of the context as well as detailed description of the phenomena of interest to allow comparisons. Many rural settings in South Asia would share the characteristics of the two study settings, although the context of urban South Asia may differ in some aspects. Detailed descriptions of context, methods and analysis enhance dependability and confirmability.

## Conclusions

Social inclusion is a critical part of a nation’s human rights framework as well as an important goal for societies that seek to promote equality and participation by all. Promoting social inclusion for PWMD requires substantial development of access to mental health services, general health services and employment opportunities. NGOs, grass-root community workers and government programmes need to work with affected communities and families to build awareness, knowledge, skills, and to ensure access to effective care as well as expanding opportunities for PWMD themselves to participate as active agents for their own well-being and livelihood.

This study presents the experiences of exclusion and inclusion for PWMD in two rural communities in North India. We found that PWMD can walk through their community and encounter ridicule, discrimination and loss of social relationships. We also found that some PWMD continue to be trusted with responsibility for household finances, income for the family and are able to maintain positive social relationships. The banyan tree typically stands sentinel in most Indian villages, a large umbrella of branch and leaf, with shafts of sunlight filtering through. Like most facets of human experience, inclusion and exclusion are a chaotic mix of light and shadow as complex as the dappled shade beneath a banyan tree.

## Abbreviations

PWMD: People with mental disorder
HIC: High income country
EHA: Emmanuel Hospital Association
LMIC: Low middle income country
